# Accidental epidural catheter removal rates and strength required for disconnection: a retrospective cohort and laboratory study

**DOI:** 10.1186/s12871-022-01728-z

**Published:** 2022-06-16

**Authors:** Yoshiaki Ishida, Yoichiro Homma, Takashi Kawamura, Masatoshi Sagawa, Yoshie Toba

**Affiliations:** 1 Department of Anesthesiology, Seirei Hamamatsu General Hospital, 2-12-12 Sumiyoshi, Naka-ku, Hamamatsu-shi, Shizuoka, 430-8558 Japan; 2 Department of General Internal Medicine, Seirei Hamamatsu General Hospital, Hamamatsu, Japan; 3 Department of Clinical Engineering, Seirei Hamamatsu General Hospital, Hamamatsu, Japan

**Keywords:** Epidural analgesia, Epidural catheter, Accidental removal, Catheter connector, Dislodgement, Disconnection, Taping, ISO 80369–6, NRFit, Laboratory study

## Abstract

**Background:**

Epidural catheters are associated with certain risks such as accidental epidural catheter removal, including dislodgement and disconnection. Globally, neuraxial connector designs were revised in 2016 to provide new standardization aimed at decreasing the frequency of misconnections during the administration of medications. However, no studies have investigated accidental epidural catheter removal after the revised standardization. This study aimed to examine differences in dislodgement and disconnection rates associated with different catheter connector types, and to investigate the linear tensile strength required to induce disconnection.

**Methods:**

This retrospective cohort study included adult patients who underwent elective surgery and received patient-controlled epidural analgesia. Patients were divided into groups according to the type of catheter connection used: old standard, new standard, and new standard with taping groups. Furthermore, we prepared 60 sets of epidural catheters and connectors comprising 20 sets for each of the old, new, and taping groups, and used a digital tension meter to measure the maximum tensile strength required to induce disconnection.

**Results:**

This clinical study involved 360, 182, and 378 patients in the old, new, and taping groups, respectively. Dislodgement rates did not differ statistically among the three groups, while there was a significant difference in disconnection rates. Propensity score matching analysis for disconnection rates showed no difference between the old and new groups (2.8% vs. 4.5%, *p* = 0.574), while the new group had higher rates than the taping group (6.5% vs. 0%, *p* = 0.002). This laboratory study identified that a tensile strength of 12.41 N, 12.06 N, and 19.65 N was required for disconnection in the old, new, and taping groups, respectively, and revealed no significant difference between the new and old groups (*p* = 0.823), but indicated a significant difference between the new and taping groups (*p* < 0.001).

**Conclusions:**

This clinical study suggested that dislodgement rates did not change among the three groups. Both clinical and laboratory studies revealed that disconnection rates did not change between the old and new connectors. Moreover, as a strategy to prevent accidents, taping the connecting points of the catheter connectors led to an increase in the tensile strength required for disconnection.

**Supplementary Information:**

The online version contains supplementary material available at 10.1186/s12871-022-01728-z.

## Background

Epidural analgesia (EA) is effective and helps improve post-surgical morbidity and hospitalization outcomes [1–3]. EA is associated with the risk of complications and adverse events, such as accidental epidural catheter removal, including catheter dislodgement and disconnection, post-dural puncture headache, local anesthetic toxicity, and epidural abscess or hematoma formation [4]. Although accidental epidural catheter dislodgement and disconnection are minor complications, they could be associated with the catheter, connector, and filter themselves rather than patient- or staff-related factors [5–10].

For a long time, universal Luer systems have been used to securely connect fittings between needles, syringes, and tubing. These connectors serve multiple medication delivery routes, including the intravenous, enteral, neuraxial, and respiratory routes. However, Luer standardization resulted in wrong-route administration due to misconnection between the administration routes [11], increasing the potential for patient harm or death. Although, the ISO 80369 series (old standard) specified new standards in 2010 to replace the universal use of Luer connectors in an effort to create new and less error-prone systems, catheter-related accidental premature removal was reported subsequently, due to connector blockages [5–8] and disconnection between the connector and filter [9, 10].

In 2016, the international standard ISO 80369–6 (NRFit™) was published to help prevent neuraxial drug administration errors; the guideline recommended a non-Luer neuraxial connector design, which has become the new standard [12]. Thus, new technological advancements may improve patient safety by preventing misconnection events [13]. Transition to the new standard NRFit™ began in October 2019 at all hospitals in Japan. Our hospital adopted the new standard in March 2020. Subsequently, the number of reports of accidental catheter disconnection submitted to the Safety Management Committee increased. Consequently, we started taping the connecting points of the catheter connectors in two places with a loop, which appeared to reduce the frequency of accidental disconnections.

Based on these observations, we hypothesized that the use of the new standard connector increases the rates of catheter removal, and that taping reduces this rate. Therefore, we decided to investigate and compare the old and new neuraxial connector standardization methods and the taping method. In addition, this study involved a laboratory investigation of the linear tensile strength required to induce disconnection.

## Methods

### Study design and setting

The Institutional Review Board, which is also the research ethics committee, of Seirei Hamamatsu General Hospital approved this single-center retrospective cohort study (approval number: 3570; February 17, 2021) and waived the requirement for written informed consent. Study information was available on the hospital website, allowing participants the opportunity to opt‐out; patients who did not opt-out were included in this study.

In 2012, our hospital was certified by the Joint Commission International (JCI), a medical function evaluation organization that works to improve patient safety and quality of health care. Furthermore, in December 2021, we passed the fourth certification examination.

The study site is an urban tertiary acute care and teaching hospital with 750 beds and a surgical load of 11,000 patients per year, among which approximately 7,000 patients are managed by the anesthesiology department. The laboratory component of this study was performed in a designated area at the surgery center of our hospital. This manuscript adhered to the Strengthening the Reporting of Observational Studies in Epidemiology statement guidelines [14].

### Study population

All adult patients (aged ≥ 20 years) who underwent elective surgery, specifically, abdominal, orthopedic, gynecologic, thoracic, urologic, or breast surgery, and received patient-controlled EA (PCEA) for postoperative analgesia in the general ward between December 1, 2019 and August 31, 2020 were included in this study. The exclusion criteria for participation in the study were unplanned removal of the epidural catheter at the post-anesthesia care unit (PACU), reoperation required during PCEA administration, and incomplete data. The patients were divided into three groups according to the type of catheter connection used: patients for whom the old standard was used (December 1, 2019 to February 29, 2020; old group), those for whom the new standard was used (March 1, 2020 to April 19, 2020; new group), and those for whom the new standard with a taped catheter connector connection was used (April 20, 2020 to August 31, 2020; taping group).

### Data collection and outcome variables

Data on the patients’ demographic and clinical characteristics, including preoperative morbidities, operation type, intraoperative anesthesia information, postoperative states, and accidental epidural catheter dislodgement and disconnection, were extracted from electronic medical records. The variables of interest included age, sex, height, weight, and body mass index (BMI). We included the American Society of Anesthesiologists Physical Status (ASA-PS) grade among preoperative morbidities. Abdominal surgeries included upper gastrointestinal surgery, hepato-biliary-pancreatic surgery, and colorectal surgery; orthopedic surgery included lower limb orthopedic surgery. We identified the interspace level of epidural catheter placement from the intraoperative anesthesia information and divided the patients into upper and middle thoracic vertebrae (Th3-7), lower thoracic vertebrae (Th8-12), and lumbar spine (L1-4) groups on the basis of this information. Among postoperative states, we recorded the duration of EA. Although the incidence of dementia and postoperative delirium as preoperative and postoperative morbidities, respectively, may be associated with accidental epidural catheter dislodgement and disconnection, these historical data are usually considered unreliable in the absence of neurological evaluations. Therefore, we did not extract these historical data.

**Fig. 1:**
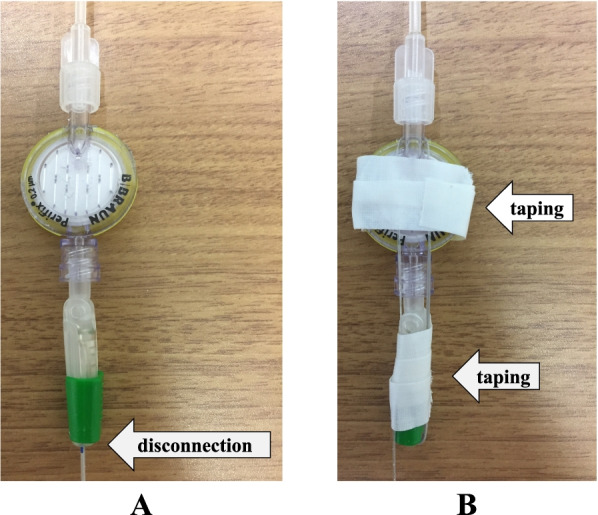
Type of catheter removal and epidural catheter connection. **A** Disconnection was defined as catheter removal at the catheter connector connection site. **B** Taping involved looping around the epidural catheter that was secured to the connector and filter in two places using surgical tape. The taping wrapped around the filter plays two roles to minimize the risk when the tape wrapped around the connector is removed and to prevent the catheter wrapped around the filter from being caught on something

Accidental epidural catheter removal was categorized as premature epidural catheter dislodgement and disconnection. Dislodgement was defined as accidental and unscheduled catheter removal from the catheter insertion site, and disconnection was defined as accidental and unscheduled catheter removal at the catheter connector connection site (Fig. 1A). When the epidural catheter was removed for reasons other than dislodgement and disconnection, it was defined as “scheduled removal”. The primary outcomes included the differences in dislodgement and disconnection rates associated with the three different types of catheter connector. When a significant difference was detected in dislodgement and/or disconnection rates, we investigated the difference in the rates between the old and new groups, and the new and taping groups, as the secondary outcome.

### Epidural catheter procedure

**Fig. 2:**
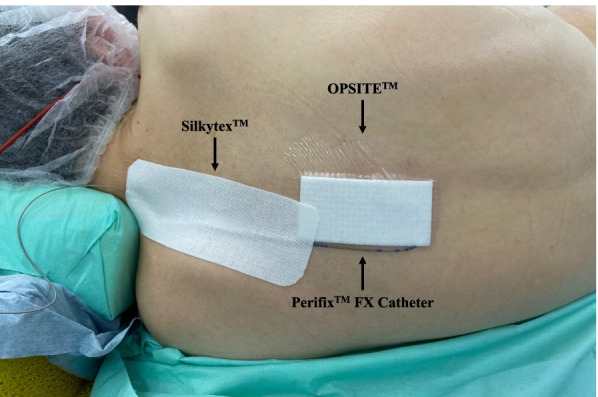
Back-fixation method. The epidural catheter is covered at the insertion site with a transparent semipermeable sterile adhesive dressing, and the rest of the catheter is secured from the dressing site to the shoulder using an elastic adhesive bandage

According to institutional standards, each anesthesiologist performed epidural puncture using a midline or paramedian approach in the lateral decubitus position and considered the interspace level of epidural catheter placement suitable for each type of surgery. Routinely, the distal end of the epidural catheter (Perifix™ FX Catheter, B. Braun, Tochigi, Japan) was inserted 3–5 cm into the epidural space. The proximal end was connected to a connector (Perifix™ Catheter Connector, B. Braun, Tochigi, Japan) and reinforced with the provided green cap. The connector was then connected to a bacterial filter (Perifix™ Filter 0.2 µm, B. Braun, Tochigi, Japan). Subsequently, the catheter was covered at the insertion site with a transparent semipermeable sterile adhesive dressing (OPSITE™ POST-OP, Smith + Nephew, Tuttlingen, Germany), and the rest of the catheter was secured from the dressing site to the shoulder using an elastic adhesive bandage (Silkytex™ White No. 5, ALCARE, Tokyo, Japan) (Fig. 2). We did not fix the catheter at the injection site with thread or tape. All patients received a local bolus anesthetic dose or continuous infusion through the epidural catheter during surgery at the discretion of the anesthetist.

### Old, new, and taping methods

In the old and new methods, the connector is only reinforced with the green cap provided with the device (Fig. 1A). The taping method entailed forming a loop around the epidural catheter, hooking the loop on the filter and securing the catheter in two places on the connector and filter using surgical tape (NICHIBAN™ < For hospitals > , NICHIBAN, Tokyo, Japan) (Fig. 1B). The three methods are summarized in Table 1.

**Table 1:** Old, new and taping methods

	Old method	New method	Taping method
Green cap	Used	Used	Used
Type of catheter connection	Old standard	New standard	New standard
Taping	Not used	Not used	Used
Appearance	Figure 1A	Figure 1A	Figure 1B

### Postoperative epidural catheter management

At the end of surgery, the filter was connected to the infusion route of a 300-mL or 150-mL PCEA pump, which was aseptically conditioned with 100–300 mL of analgesics. Patients were transferred to the PACU, where their pain scores and levels of motor block were assessed by the nursing staff and the attending anesthesiologist. Motor impairment, as a drug-associated adverse event, was managed by a temporary reduction or discontinuation of the infusion. Epidural catheters suspected of being inserted into the spinal subarachnoid space were removed immediately; these cases were excluded from analysis. In the absence of adverse events in the PACU, the patient was transferred to the general ward. The timing of epidural catheter removal was determined by the attending physician and their team responsible for postoperative management in the general ward, including the administration of postoperative analgesia. When accidental epidural catheter removal was reported either by a nurse or patient, the physician on duty responded, as required, including removing the remaining catheter.

The Safety Management Committee in our hospital requires all staff to report incidents and accidents. Therefore, we judged that catheters were removed as scheduled according to postoperative courses established by each department for cases that lacked reports on accidental catheter removal within the medical records.

### Product preparation

We prepared 20 sets of standard connectors (Perifix™ Catheter Connector, B. Braun, Tochigi, Japan) and filters (Perifix™ Filter 0.2 µm, B. Braun, Tochigi, Japan), 40 sets of new standard connectors and filters, and 60 sets of epidural catheters (Perifix™ Catheter Connector, B. Braun, Tochigi, Japan) and the provided green caps. Moreover, 20 sets of the new standard connectors involved a loop formed around the epidural catheter that secured it to the connector and filter using surgical tape (NICHIBAN™ < For hospitals > , NICHIBAN, Tokyo, Japan). One anesthesiologist (YI) assembled the 60 sets of epidural catheters, connectors, and filters as per standard clinical practice. The 60 sets were grouped and compared according to the catheter connector connection type into the old, new, and taping groups. We also prepared a digital tension meter (Digital Force Gauge™ DS2-200 N, IMADA, Aichi, Japan) to measure the tensile strength of the catheter and connector, as well as a hook linking the filter and tension meter.

### Measuring methods

**Fig. 3:**
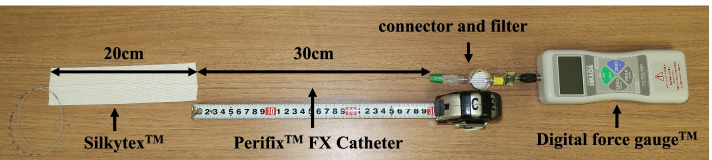
Measurement conditions. The epidural catheter is secured to the desk with a 20 cm elastic adhesive bandage and fixed 30 cm away from the connector

One clinical engineer (TK) secured the epidural catheter to the desk with 20 cm of elastic adhesive bandage (Silkytex™ White No. 5, ALCARE, Tokyo, Japan) (Fig. 3). The epidural catheter was fixed 30 cm away from the connector and supported to remain in place. Another clinical engineer (MS) slowly and linearly pulled the tension meter connected to the epidural set at a constant velocity until disconnection was achieved. We did not assume a specific clinical situation and measured the maximum tensile strength required to induce disconnection according to previously established methodology [15, 16]. We investigated the linear tensile strength required to induce disconnection as the secondary outcome.

### Statistical analyses

This was an exploratory study to examine the differences in accidental catheter removal rates associated with different catheter connector types. Therefore, we could not perform an estimation of sample size or power calculation in advance. Hence, the sample size was based on the data available during the study period. Moreover, because the period during which the new connector being used was fixed, the number of cases in the new group was used as the baseline, and the study period was set to allow the number of patients in the old and taping groups to be twice that in the new group to maximize the power (1-β).

Continuous variables are presented as the mean with standard deviation (SD). Graphical methods were used to confirm the normal distribution of the variables. Comparisons among the groups were performed using the t-test for variables that were normally distributed. Categorical variables are reported as counts (%); comparisons among groups were performed using Fisher’s exact test.

Residual analysis was used to compare the real and expected values, derived from the removal method. We presented the adjusted residual, which is a suitable statistic for comparisons among the three groups instead of confidence intervals (CIs). When significant differences were detected in the results of the residual analysis, we conducted propensity score matching (PSM) analysis as a *post-hoc* test between the old and new groups, and the new and taping groups to reduce the effects of covariates. The covariates selected for PSM analysis were factors that are considered clinically important, including age, sex, height, BMI, and ASA-PS grade. The nearest-neighbor matching method (1:1 ratio) was used, with a caliper width of 0.2 for the logit-transformed propensity score. The standardized mean difference (SMD) was examined to determine the balance between groups, and an SMD of < 0.2 was considered indicative of balance. Collinearity among covariates was assessed using the variance inflation factors. The adjusted 95% confidence intervals were calculated.

We conducted the laboratory study and statistical analyses based on the assumption that commonly manufactured products have a certain degree of normal distribution and uniformity because non-uniform products are generally removed during the manufacturing process; thus, we assumed that the data were normally distributed with homogeneous variance. The groups were compared using one-way analysis of variance. Moreover, Dunnett's multiple comparisons test was performed as a *post-hoc* test, using the new group as the control group. Based on previous studies [15, 16], the sample size was 20 tests per group.

Statistical analyses were performed using EZR version 1.54 (Saitama Medical Center, Jichi Medical University, Saitama, Japan) [17]. P-values of < 0.05 were considered statistically significant (α). The co-author performing the analysis (YH) was blinded to the study outcomes and group assignment.

## Results

### Baseline characteristics of participants

**Fig. 4:**
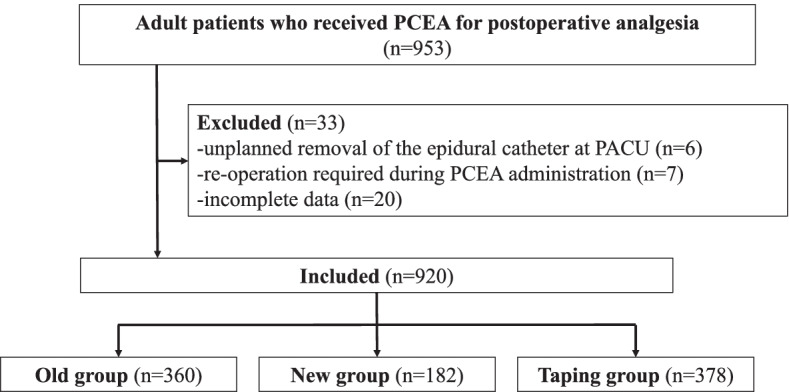
Flowchart of the study

In total, 953 patients received PCEA for postoperative analgesia; among them, 13 were excluded due to unplanned catheter removal and re-operation. Twenty additional patients were excluded for incomplete data and were considered missing completely at random (MCAR). Finally, 920 patients were included in this study, and they were allocated into either the old (*n* = 360), new (*n* = 182), or taping (*n* = 378) groups based on the type of catheter connection used (Fig. 4). The patients’ characteristics and perioperative findings are summarized in Table 2. The mean (SD) duration of EA was 2.44 (1.13) vs. 2.51 (0.98) vs. 2.66 (1.15) days for the old, new, and taping groups (*p* = 0.07), respectively.

**Table 2:** Baseline characteristics of patients grouped by type of catheter connection

	Old group	New group	Taping group
	(*n* = 360)	(*n* = 182)	(*n* = 378)
Age (years)	56.1 (15.8)	56.6 (15.5)	64.1 (14.3)
Sex (male)	102 (28.3%)	49 (26.9%)	193 (51.1%)
Height (cm)	158.8 (8.4)	159.5 (7.6)	160.0 (9.0)
Weight (kg)	58.5 (11.8)	59.5 (12.4)	59.6 (12.2)
BMI (kg/m^2^)	23.2 (3.9)	23.4 (4.0)	23.3 (4.0)
ASA-PS			
1	98 (27.2%)	40 (22.0%)	58 (15.3%)
2	216 (60.0%)	123 (67.6%)	256 (67.7%)
3	46 (12.8%)	19 (10.4%)	64 (16.9%)
Surgery type			
Abdominal	130 (36.1%)	62 (34.1%)	192 (50.8%)
Breast	2 (0.6%)	5 (2.7%)	5 (1.3%)
Gynecology	144 (40.0%)	73 (40.1%)	36 (9.5%)
Lower orthopedic	54 (15.0%)	20 (11.0%)	67 (17.7%)
Thoracic	30 (8.3%)	12 (6.6%)	42 (11.1%)
Urology	0 (0.0%)	10 (5.5%)	36 (9.5%)
Interspace level			
A: Th3/4-Th7/8	36 (10.0%)	15 (8.2%)	48 (12.7%)
B: Th8/9-Th12/L1	267 (74.2%)	143 (78.6%)	261 (69.0%)
C: L1/2-L4/5	57 (15.8%)	24 (13.2%)	69 (18.3%)

Values are presented as means (SDs) or counts (%)

Abbreviations: *BMI* body mass index, *ASA-PS* American Society of Anesthesiologists Physical Status

### Rates of accidental epidural catheter dislodgement and disconnection as the primary outcome

Fisher’s exact test was performed for comparison among the three groups (Cramer’s V = 0.103, *p* < 0.001; Table 3). A total of 10 cases of accidental epidural catheter dislodgement were observed, 6 patients (1.7%, adjusted residual = 1.360), 1 patient (0.5%, adjusted residual = -0.781), and 3 patients (0.8%, adjusted residual = -0.717) were in the old, new, and taping groups, respectively. There was no difference in dislodgement rates among the groups. In contrast, a total of 18 cases of accidental epidural catheter disconnection were observed, 7 patients (1.9%, adjusted residual = -0.021), 10 patients (5.5%, adjusted residual = 3.848), and 1 patient (0.3%, adjusted residual = -3.094) were in the old, new, and taping groups, respectively. There was a difference in disconnection rates among the groups, and the rates were the highest in the new group and the lowest in the taping group.

**Table 3:** Rates of accidental epidural catheter removal by type of catheter connection

Removal method	Old group	New group	Taping group
	(*n* = 360)	(*n* = 182)	(*n* = 378)
Scheduled			
Count	347 (96.4%)	171 (94.0%)	374 (98.9%)
Adjusted residual	-0.804	-2.631	2.927
Dislodgement			
Count	6 (1.7%)	1 (0.5%)	3 (0.8%)
Adjusted residual	1.360	-0.781	-0.717
Disconnection			
Count	7 (1.9%)	10 (5.5%)	1 (0.3%)
Adjusted residual	-0.021	3.848	-3.094

Values are presented as counts (%) or adjusted residuals

Residual analysis was based on Fisher's exact test

Adjusted residuals smaller than − 1.96 or greater than 1.96 indicate a statistically significant difference at *p* < 0.05

Adjusted residuals smaller than − 2.58 or greater than 2.58 indicate a statistically significant difference at *p* < 0.01

### PSM analysis as the secondary outcome

PSM analysis of the old and new groups matched 181 patients per group. The groups were matched for age, sex, height, BMI, and ASA-PS grade and were well-balanced with an SMD < 0.2. There was no difference in the rates of premature epidural catheter disconnection between the groups (2.8% vs. 5.5%, 95% CI = 0.128–1.602, *p* = 0.292; Table 4). In contrast, PSM analysis of the new and taping groups matched 162 patients per group. The groups were matched for age, sex, height, BMI, and ASA-PS grade and were well-balanced with an SMD < 0.2. Disconnection rates were higher in the new group than in the taping group (6.2% vs. 0%, 95% CI = 0.000–0.429, *p* = 0.002; Table 5).

**Table 4:** Propensity score matching between the old and new catheter groups

	Unadjusted values			After propensity score matching			
	Old group	New group	*P*-value	Old group	New group	*P*-value	SMD
	(*n* = 354)	(*n* = 181)		(*n* = 181)	(*n* = 181)		
Age (years)	55.9 (15.9)	56.6 (15.5)	0.658	56.7 (15.8)	56.6 (15.5)	0.941	0.008
Sex (male)	101 (28.5%)	49 (27.1%)	0.761	45 (24.9%)	49 (27.1%)	0.719	0.050
Height (cm)	158.9 (8.5)	159.5 (7.7)	0.355	158.9 (8.1)	159.5 (7.7)	0.422	0.085
Weight (kg)	58.5 (11.9)	59.6 (12.3)	0.326	59.5 (12.8)	59.6 (12.3)	0.920	0.011
BMI (kg/m^2^)	23.2 (3.9)	23.4 (4.0)	0.546	23.6 (4.3)	23.4 (4.0)	0.753	0.033
ASA-PS							
1	98 (27.7%)	40 (22.1%)	0.238	36 (19.9%)	40 (22.1%)	0.880	0.055
2	212 (59.9%)	122 (67.4%)		126 (69.6%)	122 (67.4%)		
3	44 (12.4%)	19 (10.5%)		19 (10.5%)	19 (10.5%)		
Surgery type							
Abdominal	126 (35.6%)	62 (34.3%)	N/A	62 (34.3%)	62 (34.3%)	0.005	0.420
Breast	2 (0.6%)	5 (2.8%)		1 (0.6%)	5 (2.8%)		
Gynecology	143 (40.4%)	73 (40.3%)		73 (40.3%)	73 (40.3%)		
Lower orthopedic	54 (15.3%)	19 (10.5%)		29 (6.0%)	19 (10.5%)		
Thoracic	29 (8.2%)	12 (6.6%)		16 (8.8%)	12 (6.6%)		
Urology	0 (0.0%)	10 (5.5%)		0 (0%)	10 (5.5%)		
Interspace level							
A: Th3/4-Th7/8	34 (9.6%)	15 (8.3%)	0.484	19 (10.5%)	15 (8.3%)	0.288	0.169
B: Th8/9-Th12/L1	263 (74.3%)	143 (79.0%)		130 (71.8%)	143 (79.0%)		
C: L1/2-L4/5	57 (16.1%)	23 (12.7%)		32 (17.7%)	23 (12.7%)		
Removal method							
Scheduled	347 (98.0%)	171 (94.5%)	0.036	176 (97.2%)	171 (94.5%)	0.291	0.139
Disconnection	7 (2.0%)	10 (5.5%)		5 (2.8%)	10 (5.5%)		

Abbreviations: *BMI* body mass index, *ASA-PS* American Society of Anesthesiologists Physical Status, *SMD* standardized mean difference

**Table 5:** Propensity score matching between the new and taping catheter groups

	Unadjusted values			After propensity score matching			
	New group	Taping group	*P*-value	New group	Taping group	*P*-value	SMD
	(*n* = 181)	(*n* = 375)		(*n = *162)	(*n* = 162)		
Age (years)	56.6 (15.5)	64.0 (14.3)	< 0.001	58.5 (14.9)	58.2 (14.5)	0.853	0.021
Sex (male)	49 (27.1%)	191 (50.9%)	< 0.001	49 (30.2%)	57 (35.2%)	0.407	0.105
Height (cm)	159.5 (7.7)	160.0 (9.0)	0.543	159.3 (8.0)	160.4 (8.3)	0.204	0.141
Weight (kg)	59.6 (12.3)	59.7 (12.2)	0.956	59.0 (12.1)	59.2 (12.2)	0.894	0.015
BMI (kg/m^2^)	23.4 (4.0)	23.3 (4.0)	0.782	23.3 (3.8)	23.0 (4.3)	0.660	0.049
ASA-PS							
1	40 (22.1%)	58 (15.5%)	0.037	34 (21.0%)	30 (18.5%)	0.519	0.130
2	122 (67.4%)	253 (67.5%)		109 (67.3%)	118 (72.8%)		
3	19 (10.5%)	64 (17.1%)		19 (11.7%)	184(8.6%)		
Surgery type							
Abdominal	62 (34.3%)	189 (50.4%)	N/A	61 (37.7%)	69 (42.6%)	0.003	0.473
Breast	5 (2.8%)	5 (1.3%)		2 (1.2%)	4 (2.5%)		
Gynecology	73 (40.3%)	36 (9.6%)		58 (35.8%)	27 (16.7%)		
Lower orthopedic	19 (10.5%)	67 (17.9%)		19 (11.7%)	32 (19.8%)		
Thoracic	12 (6.6%)	42 (11.2%)		12 (7.4%)	18 (11.1%)		
Urology	10 (5.5%)	36 (9.6%)		10 (6.2%)	12 (7.4%)		
Interspace level							
A: Th3/4-Th7/8	15 (8.3%)	48 (12.8%)	0.043	14 (8.6%)	22 (13.6%)	0.112	0.237
B: Th8/9-Th12/L1	143 (79.0%)	258 (68.8%)		125 (77.2%)	108 (66.7%)		
C: L1/2-L4/5	23 (12.7%)	69 (18.4%)		23 (14.2%)	32 (19.8%)		
Removal method							
Scheduled	171 (94.5%)	374 (99.7%)	< 0.001	152 (93.8%)	162 (100%)	0.002	0.363
Disconnection	10 (5.5%)	1 (0.3%)		10 (6.2%)	0 (0%)		

Abbreviations: *BMI* body mass index, *ASA-PS* American Society of Anesthesiologists Physical Status, *SMD* standardized mean difference

### Laboratory study results as the secondary outcome

**Fig. 5:**
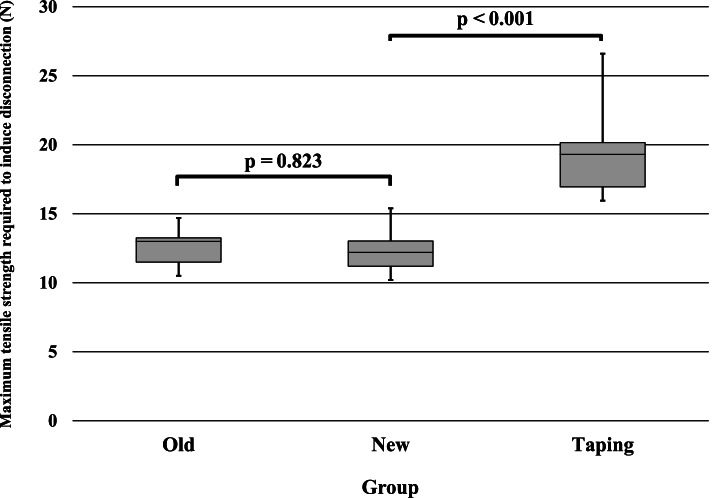
Box plot of tensile strength values. In this box plot, a box spans from the first quartile to the third quartile, and the whiskers indicate the distance from each quartile to the minimum or maximum. The old and new groups used the standard and new connectors, respectively, and filters. The taping group used the new connector and involved looping around the epidural catheter, which is secured to the connector and filter using surgical tape

The mean (SD) tensile strength was calculated for each group; there was a significant difference among the groups in the tensile strength required for disconnection (*p* < 0.001). The highest tensile strength values were recorded for the taping group, with a mean value of 19.65 (3.19) N. The corresponding values for the old and new groups were 12.41 (1.16) N and 12.06 (1.40) N, respectively (Fig. 5).

Dunnett's multiple comparison test revealed that the tensile strength values recorded for the old and new groups were comparable (mean difference = 0.35, *p* = 0.823), and that there was a significant difference in the tensile strength recorded for the new and taping groups (mean difference = 7.59, *p* < 0.001).

## Discussion

This was a retrospective cohort study examining differences in accidental catheter removal rates associated with different catheter connector types. Overall, 28 of 920 (3.0%) patients experienced accidental epidural catheter removal; among them, dislodgement and disconnection occurred in 10 (1.09%) and in 18 (1.96%) patients, respectively. In addition, the laboratory component of our study revealed that greater tensile strength was required for disconnection in the taping group as compared with the other groups.

Incomplete data in this study included information on patients’ height and weight and the interspace level of epidural catheter placement. Our hospital has cleared the JCI standard that is required to ensure the quality of medical records. Therefore, our staff is trained and undergoes daily checks to ensure the quality of medical records. In summary, we consider it appropriate to assume that the incomplete data in this study are based on MCAR and that their exclusion from statistical analysis is permissible.

Accidental epidural catheter dislodgement may result in inadequate analgesia, leading to the occurrence of complications or extension of hospital stay. Therefore, various methods of reducing the risk of dislodgement have been proposed, which include the standard use of steri-strips and clear adhesive dressing, as well as the Lockit design [18], attaching the catheter to the skin with dressing and a single suture [19], and using a tunneling technique [20]. In this study, we empirically combined the transparent semipermeable sterile adhesive dressing and elastic adhesive bandage to strengthen the fixation of the epidural catheter; fixing the catheter to the skin with transparent adhesive dressing may help prevent it from slipping.

We performed Fisher’s exact test to examine the association between dislodgement rates and different catheter connector types among the three groups. Because no prior data were available for the primary outcomes of this study, we could not calculate the sample size a priori. Additionally, we did not perform *post-hoc* power analysis since an existing database was used [21].

The lack of significant difference in dislodgement rates among the three groups may be attributed to the small sample size or low incidence rates, which could have prevented the detection of a clinically meaningful increase in the risk of dislodgement. Another reason for our findings may be the fact that the back-fixation method remained the same, despite a change in the design standards of catheter connectors. Previous studies have reported dislodgement rates in the range of 1.2–5.1% [4, 22, 23]. The dislodgement rate of 1.09% seen in this study was comparable with or lower than previously reported results, suggesting that this approach may be beneficial. However, this finding is considered exploratory at this point, and future, larger studies are necessary to further elucidate risk factors associated with accidental epidural catheter dislodgement.

In contrast, accidental epidural catheter disconnection from its connector may result in patient harm, including not only inadequate analgesia but also increased risk of bacterial contamination [24]; therefore, disconnection should be prevented whenever possible. Although product manufacturers have the responsibility to pursue design solutions that minimize the risk of such events [10], healthcare workers should supplement these efforts to ensure patient safety. A similar taping method involving the formation of a loop around the epidural catheter, which was taped to the filter alone, was introduced previously to protect the junction between the catheter and filter from the effect of any force [25]; however, we found this method ineffective in our setting and thus developed an alternative approach that involves taping in two places with a loop.

Herein, the taping wrapped around the filter plays two roles to minimize the risk when the tape that is wrapped around the connector is removed and to prevent the catheter wrapped around the filter from being caught on something. It is noteworthy that the length of the catheter fixed to the patient's back will be longer if epidural catheter placement is at the lumbar spine level (L1–4). Therefore, a shorter catheter length in the unfixed area in taller patients would be a disadvantage. However, there were no particular complaints associated with the taping method from healthcare workers and patients.

Previous studies have reported disconnection rates in the range of 1.7–2.3% [4, 22, 23]; these rates were comparable with the rate in this study’s old group, which was 1.9%. In this clinical study, there was a significant difference in disconnection rates among the three groups. Therefore, considering that some baseline variables that might influence accidental epidural catheter disconnection varied during the study period, we performed PSM analysis as a *post-hoc* test between the old and new groups, and between the new and taping groups to minimize the confounding effects due to non-randomized assignment. The results of PSM analysis of the old and new groups revealed no difference in disconnection rates. In contrast, PSM analysis of the new and taping groups indicated that disconnection occurred less frequently in the taping group. In this laboratory study, there was no significant difference in the tensile strength required for disconnection between the new and old groups, but there was a significant difference between the new and taping groups.

Findings from both clinical and laboratory trials suggest that disconnection rates did not vary between the old and new connectors and that taping the connecting points of the catheter connectors in two places with a loop may reduce the risk of disconnection. Due to the efforts of product manufacturers, connectors that do not comply with international standards cannot be used in the future, thereby effectively reducing drug administration errors. For the safety of patients, however, we as healthcare workers consider it important to devise strategies to prevent accidents in our daily work and to compare and verify the safety of products as in this study.

To the best of our knowledge, this is the first study examining accidental catheter removal rates and connection strength of epidural catheter connectors since the use of epidural connectors and filters has become the international standard. Several previous studies have investigated accidental removal rates of epidural catheters [4, 22, 23, 26, 27]; however, few clinical studies have directly compared epidural connector designs. Doyle et al. [15] compared the connection strength of epidural catheter connectors using increment weight as a surrogate measure of linear force. Richardson et al. [16] compared the connection strength using dynamic linear force testing under controlled laboratory conditions. In contrast, we investigated the connection strength of epidural catheter connectors, including that which is compliant with the international standard published in 2016, using linear tensile strength to induce disconnection. The present findings provide evidence that may help improve patient safety.

### Limitations

This study has some limitations. First, this was a retrospective study, and there may be several known and unknown confounders, including the timing of ambulation, patient mobilization, context of accidental epidural catheter removal, incidence of dementia and postoperative delirium, and failure to record the outcome, which may have affected the present findings. Nevertheless, the present findings remain meaningful, as randomized controlled trials are impractical in this context. Furthermore, the included groups were heterogenous owing to the differences in the timing and type of surgery. We note that in April 2020, the Japanese government issued a state of emergency owing to the coronavirus disease pandemic, which resulted in the postponement of many elective surgeries [28]. Second, the epidural set used at our hospital was one of the many types available. For example, although B Braun only manufactures one type of filter and connector, three types of epidural catheters are available through this manufacturer. Therefore, the present findings may not be generalizable to catheters produced by other manufacturers. However, they may provide clinical guidance on preventing accidental epidural catheter removal, as the type of epidural catheter does not affect the back-fixation method. Finally, we were unable to perform blinded laboratory‐based testing under formal conditions, controlling for temperature, pressure, and humidity. However, the present experiment was conducted under conditions representative of those encountered in surgical practice. Furthermore, although the clinical engineer involved in the experiment pulled the tension meter connected to the epidural set at a constant velocity, it remains unclear whether the force applied was indeed constant. Finally, although the taping method may not have been applied uniformly in all cases, any variability is representative of clinical practice.

## Conclusions

The results of this clinical study suggest that dislodgement rates may not vary among the three groups. In addition, comparison and verification of the safety of products in both clinical and laboratory studies revealed that disconnection rates did not differ between the old and new connectors. Furthermore, as a strategy to prevent accidents in daily work, we found that taping the connecting points of the catheter connectors in two places with a loop led to an increase in the tensile strength required for disconnection, which may reduce the risk of disconnection in PCEA. Further studies are required to clarify other parameters that may affect patient safety.

## Supplementary Information

**Additional file 1.** 

**Additional file 2.** 

## Data Availability

The datasets used and/or analyzed during the current study are available from the corresponding author on reasonable request.

## Abbreviations

EA: Epidural Analgesia
JCI: Joint Commission International
PCEA: Patient-Controlled Epidural Analgesia
PACU: Post-Anesthesia Care Unit
BMI: Body Mass Index
ASA-PS: American Society of Anesthesiologists Physical Status
SD: Standard Deviation
CI: Confidence Interval
PSM: Propensity Score Matching
SMD: Standardized Mean Difference
MCAR: Missing Completely at Random
